# Blue Light and Methyl Jasmonate Synergistically Enhance Betalain Accumulation, Antioxidant Enzyme Activity, and Osmotic Adjustment in Sugar Beet (*Beta vulgaris* L.) Seedlings: A Time-Course Analysis

**DOI:** 10.3390/plants15131994

**Published:** 2026-06-27

**Authors:** Hui Wang, Chao Yang, Yanling Yu, Dayou Cheng, Cuihong Dai, Chengfei Luo

**Affiliations:** 1 School of Chemistry and Chemical Engineering, Harbin Institute of Technology, Harbin 150001, China; hgdcdy@126.com (D.C.); cfluo7375@hit.edu.cn (C.L.); 2 School of Astronautics, Harbin Institute of Technology, Harbin 150001, China; 15201702938@163.com; 3 School of Medicine and Health, Harbin Institute of Technology, Harbin 150001, China; dch@hit.edu.cn

**Keywords:** sugar beet, betalain, blue light, MeJA, antioxidant enzymes, proline

## Abstract

Betalains are natural antioxidant pigments valued as food colorants (E162), yet their combined responses to light quality and methyl jasmonate (MeJA) during long-term growth remain poorly understood. In this study, the interactive effects of blue light and MeJA on growth, betacyanin, betaxanthin, antioxidant enzyme activities [peroxidase (POD), superoxide dismutase (SOD), catalase (CAT)], malondialdehyde (MDA), and proline (PRO) were investigated at weeks 3, 10, and 13 of sugar beet seedling growth. Four treatments were established: white light (W, control), white light + MeJA (WM), white light + blue light (WB), and white light + blue light + MeJA (WBM). The results showed that WB rapidly induced betaxanthin accumulation and enhanced SOD and POD activities while reducing MDA at week 3. MeJA alone triggered an explosive increase in betacyanin (45.74 mg·g^−1^ FW) at week 10, accompanied by elevated activities of POD, SOD, and CAT. The combined treatment (WBM) maintained the highest betacyanin (36.48 mg·g^−1^ FW) and betaxanthin (8.97 mg·g^−1^ FW) contents, the lowest MDA level (17.17 nmol·g^−1^ FW), and a high proline level (528.39 μg·g^−1^ FW) at week 13, providing sustained pigment maintenance at the late stage. The three antioxidant enzymes exhibited a temporal division of labor: high SOD activity at the early stage, while POD and CAT activities continuously increased during the middle and late stages. Notably, the correlation between proline and MDA shifted from positive at week 10 to negative at week 13, suggesting a temporal transition in the protective role of proline against membrane lipid peroxidation during late-stage development. In conclusion, blue light and MeJA enhance the antioxidant capacity of sugar beet seedlings through a temporally synergistic pattern of “priming by blue light, burst by MeJA, and maintenance by combined treatment,” offering a potential preharvest regulation strategy for the production of natural pigments and functional food ingredients.

## 1. Introduction

Betalains are water-soluble nitrogen-containing pigments, where they functionally replace anthocyanins found in most other angiosperms [1,2,3]. Comprising red-purple betacyanins and yellow-orange betaxanthins, both derived from betalamic acid [3,4], betalains serve as natural colorants (E162) and functional food ingredients owing to their antioxidant, anti-inflammatory, and antimicrobial activities [5,6,7,8,9]. Their biosynthetic pathway has also been exploited to engineer novel flower colors [10]. Sugar beet (*Beta vulgaris* L.) is a major natural source of betalains and an established model for studying their regulation by environmental and hormonal cues [11,12].

Light quality is a key determinant of plant secondary metabolism, including betalain biosynthesis [13]. Blue light (400–500 nm) effectively induces betalain accumulation in species such as *Alternanthera brasiliana* and red beet hairy roots [14,15], while red light and UV-B radiation also promote betalain production to varying degrees, indicating species- and wavelength-dependent regulation [16,17,18]. Beyond pigment biosynthesis, light quality modulates antioxidant systems via photoreceptor-triggered reactive oxygen species (ROS) signaling: blue light induces cryptochrome-mediated ROS production and upregulates SOD, POD, and APX, whereas monochromatic red or blue light can impair high-light tolerance by compromising ROS regulation and anthocyanin stability [19,20,21]. These observations suggest that light quality influences both betalain metabolism and antioxidant defense, yet the underlying physiological mechanisms—particularly their temporal dynamics over prolonged cultivation—remain poorly characterized.

Methyl jasmonate (MeJA) is a lipid-derived defense hormone that promotes secondary metabolite accumulation across plant species. Exogenous MeJA activates the COI1-JAZ-MYC signaling cascade, leading to transcriptional upregulation of biosynthetic genes for phenolics, alkaloids, and betalains [22,23]. In betalain-producing plants, foliar application of 100 μM MeJA increased total betalain content by up to 76% in *Alternanthera* spp. [24], and similar induction has been reported in *Celosia argentea* cell cultures and red-fleshed dragon fruit [25,26]. Despite the established efficacy of MeJA as a betalain elicitor, its interaction with light quality during long-term plant development remains largely unexplored.

Crosstalk between light and jasmonate signaling is increasingly recognized as a regulatory hub integrating external cues with internal defense programs. Combined treatments of specific light qualities with MeJA often produce additive or synergistic effects exceeding either factor alone: blue light plus MeJA synergistically enhanced polyphenol accumulation in mung bean sprouts [27], and combined UV-B and MeJA increased apple anthocyanin content approximately 20-fold, whereas MeJA alone was nearly ineffective [28,29]. At the molecular level, this synergy is thought to involve the convergence of light and jasmonate pathways on transcription factors MYC2 and HY5 [30,31]. However, direct physiological evidence for such interactive effects on betalain accumulation—as opposed to anthocyanins or total phenolics—is scarce. In particular, no study has tracked betalain dynamics and associated antioxidant responses over prolonged cultivation covering the early, mid, and late vegetative stages.

To address these gaps, we conducted a 13-week time-course experiment in which sugar beet seedlings (cv. HZTC11H) were subjected to four treatments: white light control (W), white light + 150 μM MeJA (WM), white + blue light (WB), and white + blue light + 150 μM MeJA (WBM), all at a PPFD of 130 μmol·m^−2^·s^−1^. Based on the temporal profile of betalain content, weeks 3, 10, and 13 were selected as representative of the early initiation, mid-term peak, and late maintenance stages of pigment accumulation, respectively. At each time point, we measured betacyanin and betaxanthin contents, antioxidant enzyme activities (POD, SOD, CAT), malondialdehyde (MDA), and proline (PRO). We addressed three specific questions: (1) whether blue light rapidly activates betaxanthin synthesis and SOD/POD activities at the early stage; (2) whether MeJA triggers a mid-term betacyanin burst and enhances overall antioxidant defense; and (3) whether the combination of blue light and MeJA produces sustained late-stage pigment maintenance while suppressing lipid peroxidation. This design enables a systematic evaluation of the temporal interplay between light quality and MeJA in regulating betalain-related antioxidant physiology.

## 2. Results

### 2.1. Growth Phenotypes

**Figure 1:**
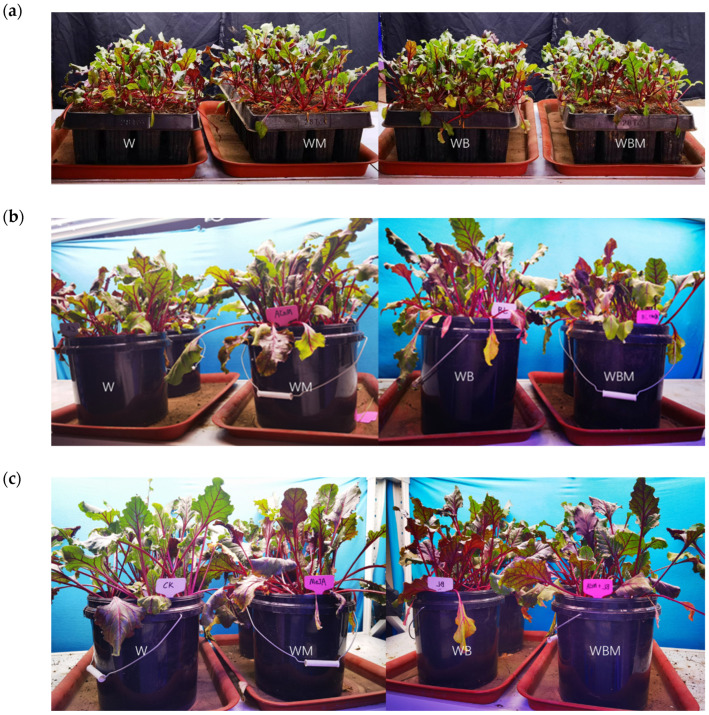
Growth phenotypes of sugar beet seedlings under different treatments. (**a**) Week 3, (**b**) Week 10, and (**c**) Week 13. From left to right within each panel, the four treatment groups are: white light control (W), white + MeJA (WM), white + blue light (WB), and white + blue light + MeJA (WBM). All treatments were maintained at a photosynthetic photon flux density (PPFD) of 130 μmol·m^−2^·s^−1^.

Representative images of sugar beet seedlings under the four treatments at weeks 3, 10, and 13 are shown in Figure 1. Visual inspection indicated that blue light-treated seedlings (WB and WBM) appeared more vigorous than those under white light alone (W and WM) at all three time points, with the WBM group showing the most pronounced improvement at weeks 10 and 13. These observations are qualitative; quantitative growth parameters were not systematically measured.

### 2.2. Betalain Accumulation

#### 2.2.1. Betacyanin Content

**Figure 2:**
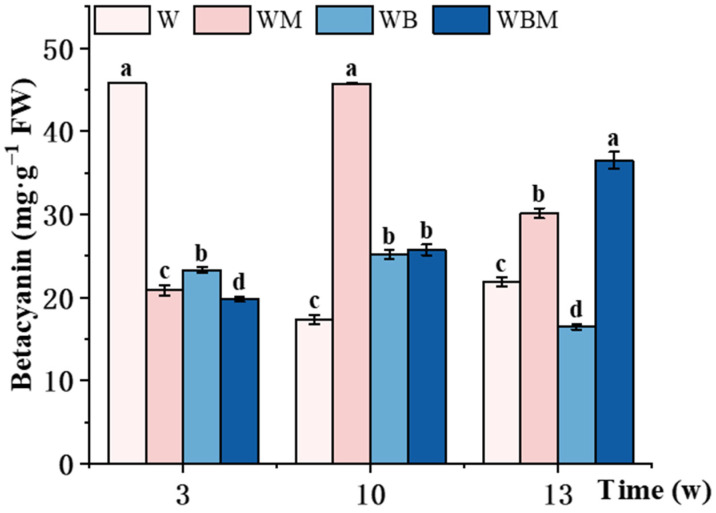
Changes in betacyanin content in sugar beet seedlings under different treatments. Note: Different lowercase letters in the figure indicate the significant differences (*p* < 0.05) between the different treatments. Vertical bars represent the standard error of means (±S.E.). Within each treatment, betacyanin content differed significantly among weeks 3, 10, and 13 (*p* < 0.05).

The betacyanin content of sugar beet seedlings under different treatments is shown in Figure 2. At week 3, the W group had the highest content (45.83 mg·g^−1^ FW), significantly exceeding the WM (20.89), WB (23.30), and WBM groups (19.79), which did not differ significantly from one another. Two-way ANOVA revealed a significant light × MeJA interaction (F_1,8_ = 2236.46, *p* < 0.001); the combined treatment (WBM) showed the lowest content, indicating an antagonistic effect at this early stage. At week 10, the WM group rose sharply to 45.74 mg·g^−1^ FW, significantly higher than the W (17.33), WB (25.19), and WBM groups (25.71). The WB and WBM groups had similar contents, both significantly higher than W. The interaction remained significant (F_1,8_ = 2284.64, *p* < 0.001), but WBM did not differ from WB and remained below WM, indicating no synergistic effect at the mid-term stage. At week 13, the WBM group reached 36.48 mg·g^−1^ FW, significantly exceeding the WB (16.44), W (21.82), and WM groups (30.11). The light × MeJA interaction was again significant (F_1,8_ = 230.62, *p* < 0.001), and Tukey’s test confirmed that WBM outperformed all other treatments (*p* < 0.05). In summary, betacyanin was highly MeJA-sensitive: WM produced the strongest mid-term burst, WB alone had no promoting effect, and WBM demonstrated synergistic enhancement specifically at week 13 (light × MeJA: F_1,8_ = 230.62, *p* < 0.001).

#### 2.2.2. Betaxanthin Content

**Figure 3:**
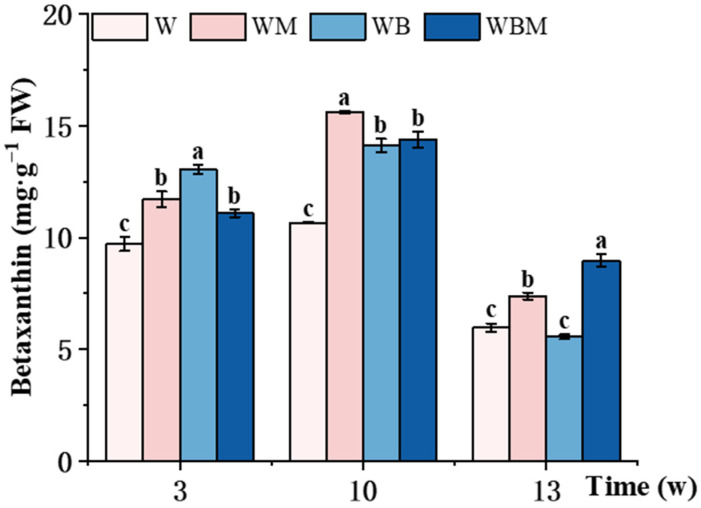
Changes in betaxanthin content in sugar beet seedlings under different treatments. Note: Different lowercase letters in the figure indicate the significant differences (*p* < 0.05) between the different treatments. Vertical bars represent the standard error of means (±S.E.). Within each treatment, betaxanthin content was significantly higher at week 10 than at weeks 3 and 13 and significantly lower at week 13 than at the two earlier time points (*p* < 0.05).

The betaxanthin content of sugar beet seedlings under different treatments is shown in Figure 3. At week 3, the WB group had the highest content (13.05 mg·g^−1^ FW), significantly higher than the W (9.70), WM (11.70), and WBM groups (11.08); WBM and WM did not differ significantly but both exceeded W. Two-way ANOVA revealed a significant light × MeJA interaction (F_1,8_ = 160.57, *p* < 0.001), and Tukey’s test confirmed the order WB > WBM ≈ WM > W. At week 10, the WM group increased to 15.61 mg·g^−1^ FW, significantly exceeding the W (10.66), WB (14.11), and WBM groups (14.40); WB and WBM did not differ but both exceeded W. The interaction remained significant (F_1,8_ = 295.71, *p* < 0.001; WM > WBM ≈ WB > W). At week 13, the WBM group reached 8.97 mg·g^−1^ FW, significantly higher than the WB (5.56), W (5.96), and WM groups (7.37), with Tukey’s test confirming WBM significantly exceeded all other treatments (*p* < 0.05; light × MeJA: F_1,8_ = 79.32, *p* < 0.001). In summary, betaxanthin responded primarily to blue light at the early stage (WB, week 3) and to MeJA at the mid-stage (WM, week 10), while the WBM combination produced the strongest synergistic effect at the late stage (week 13; light × MeJA: F_1,8_ = 79.32, *p* < 0.001).

### 2.3. Antioxidant Enzyme Activities

#### 2.3.1. POD Activity

**Figure 4:**
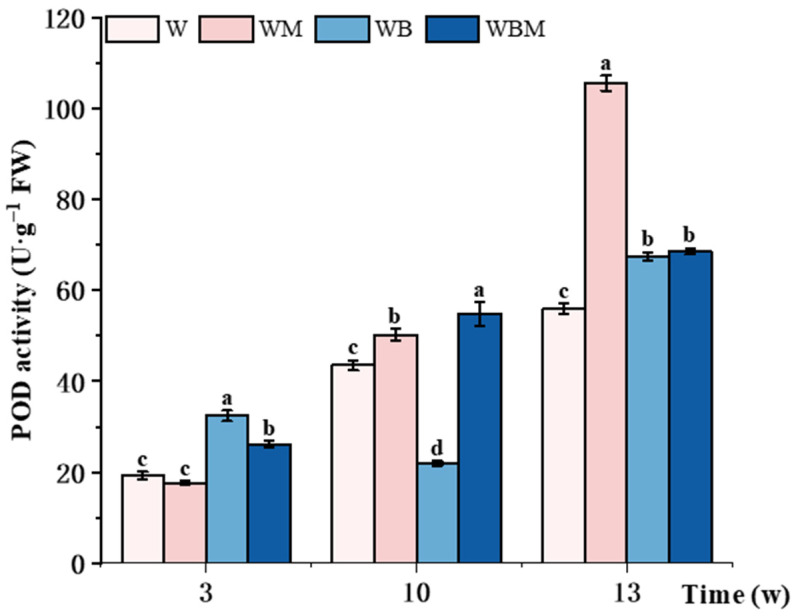
Changes in POD activity in sugar beet seedlings under different treatments. Note: Different lowercase letters in the figure indicate the significant differences (*p* < 0.05) between the different treatments. Vertical bars represent the standard error of means (±S.E.). Within each treatment, POD activity differed significantly among the three time points (*p* < 0.05).

The changes in POD activity are shown in Figure 4. At week 3, the WB group was highest (32.4 U·g^−1^ FW), significantly exceeding the W (19.3), WM (17.6), and WBM groups (26.2); WBM was significantly higher than W and WM, which did not differ. Two-way ANOVA revealed a significant light × MeJA interaction (F_1,8_ = 22.13, *p* = 0.0015; WB > WBM > W ≈ WM). At week 10, the WBM group increased to 54.9 U·g^−1^ FW, significantly higher than WB (21.9), W (43.5), and WM (50.2), indicating a positive interaction (F_1,8_ = 213.41, *p* < 0.001; WBM > WM > W > WB). At week 13, the WM group reached 105.6 U·g^−1^ FW, significantly exceeding the other three groups (W 55.9, WB 67.4, WBM 68.6); WB and WBM did not differ but both exceeded W (F_1,8_ = 1207.96, *p* < 0.001; WM > WBM ≈ WB > W). In summary, POD activity was induced early by blue light (WB, week 3) and strongly by MeJA in the late stage (WM, week 13). The WBM combination produced a transient synergistic effect at week 10 that was not sustained at week 13.

#### 2.3.2. SOD Activity

**Figure 5:**
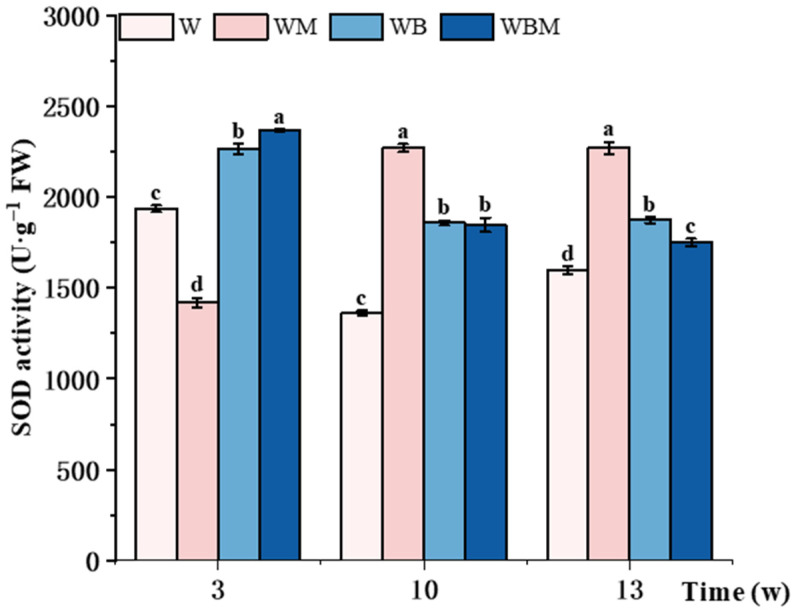
Changes in SOD activity in sugar beet seedlings under different treatments. Note: Different lowercase letters in the figure indicate the significant differences (*p* < 0.05) between the different treatments. Vertical bars represent the standard error of means (±S.E.). Within each treatment, SOD activity differed significantly among the three time points (*p* < 0.05).

The changes in SOD activity are shown in Figure 5. At week 3, the WBM group was highest (2368.17 U·g^−1^ FW), followed by WB (2262.25), both significantly exceeding W (1936.14) and WM (1419.15); Tukey’s test confirmed the order WBM > WB > W > WM (F_1,8_ = 640.05, *p* < 0.001). At week 10, WM increased to 2270.12 U·g^−1^ FW, significantly exceeding W (1362.67), WB (1858.08), and WBM (1846.81); WB and WBM did not differ but both exceeded W (F_1,8_ = 1079.79, *p* < 0.001; WM > WB ≈ WBM > W). At week 13, WM remained highest (2268.86 U·g^−1^ FW), significantly higher than W (1596.43), WB (1873.31), and WBM (1749.79), with the order WM > WB > WBM > W (F_1,8_ = 784.94, *p* < 0.001). In summary, a synergistic effect on SOD activity (WBM > WB) was observed only at the early stage (week 3). MeJA alone (WM) produced the highest activities at weeks 10 and 13, while WBM was less effective than WM and even lower than WB at week 13.

#### 2.3.3. CAT Activity

**Figure 6:**
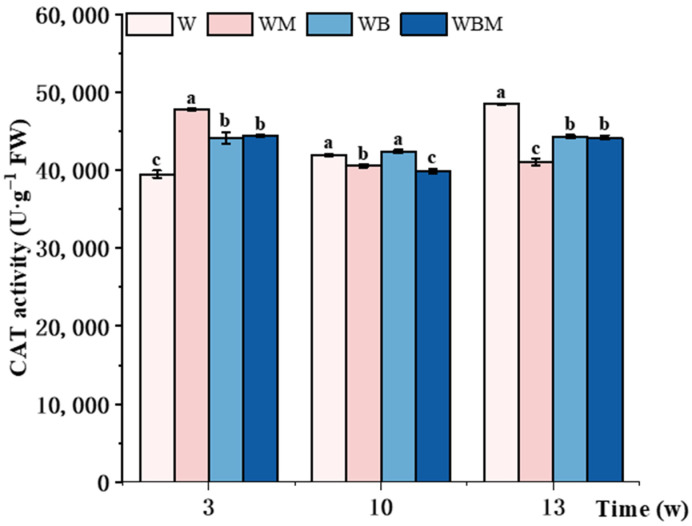
Changes in CAT activity in sugar beet seedlings under different treatments. Note: Different lowercase letters in the figure indicate the significant differences (*p* < 0.05) between the different treatments. Vertical bars represent the standard error of means (±S.E.). Within each treatment, CAT activity differed significantly among the three time points (*p* < 0.05).

The changes in CAT activity are shown in Figure 6. At week 3, the WM group was highest (47,787.96 U·g^−1^ FW), significantly exceeding W (39,497.28), WB (44,111.08), and WBM (44,405.14); WB and WBM did not differ but both exceeded W (F_1,8_ = 243.70, *p* < 0.001; WM > WBM ≈ WB > W). At week 10, WB (42,408.58) and W (41,957.34) were similar and both significantly higher than WM (40,551.26) and WBM (39,893.29), with WBM the lowest (F_1,8_ = 18.37, *p* = 0.0027; WB ≈ W > WM > WBM). At week 13, the W group reached 48,473.78 U·g^−1^ FW, significantly exceeding WB (44,302.24), WBM (44,165.36), and WM (41,044.50); WB and WBM did not differ but both exceeded WM (F_1,8_ = 565.29, *p* < 0.001; W > WB ≈ WBM > WM). In summary, MeJA transiently induced CAT activity at week 3 under white light (WM), while blue light maintained CAT at intermediate levels. Notably, WBM showed no synergistic effect on CAT activity at any time point.

### 2.4. Malondialdehyde and Proline Contents

#### 2.4.1. MDA Content

**Figure 7:**
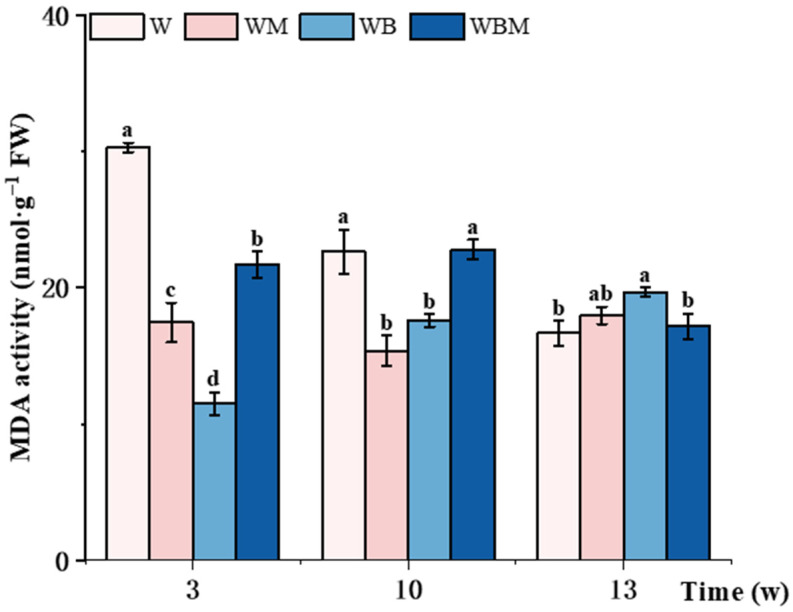
Changes in MDA content in sugar beet seedlings under different treatments. Note: Different lowercase letters in the figure indicate the significant differences (*p* < 0.05) between the different treatments. Vertical bars represent the standard error of means (±S.E.). Within the W, WB, and WBM groups, MDA content differed significantly among time points (*p* < 0.05); no significant temporal differences were detected in the WM group (*p* = 0.063).

The changes in MDA content are shown in Figure 7. At week 3, the W group had the highest MDA content (30.28 nmol·g^−1^ FW), followed by WBM (21.68), WM (17.45), and WB (11.50), with all pairwise differences significant (F_1,8_ = 412.39, *p* < 0.001; W > WBM > WM > WB). At week 10, W (22.62) and WBM (22.80) were both elevated and did not differ, while WM (15.36) and WB (17.59) were significantly lower (F_1,8_ = 98.36, *p* < 0.001; W ≈ WBM > WB ≈ WM). At week 13, WB increased to the highest level (19.68), significantly exceeding W (16.67) and WBM (17.17), while WM (17.95) did not differ significantly from any group (F_1,8_ = 19.69, *p* = 0.0022; WB > W, with WM and WBM intermediate). Overall, MDA declined progressively in the W group, increased gradually in the WB group, and remained stably low in the WM group across all time points, while WBM showed an initial increase at week 10 followed by a decline at week 13.

#### 2.4.2. PRO Content

**Figure 8:**
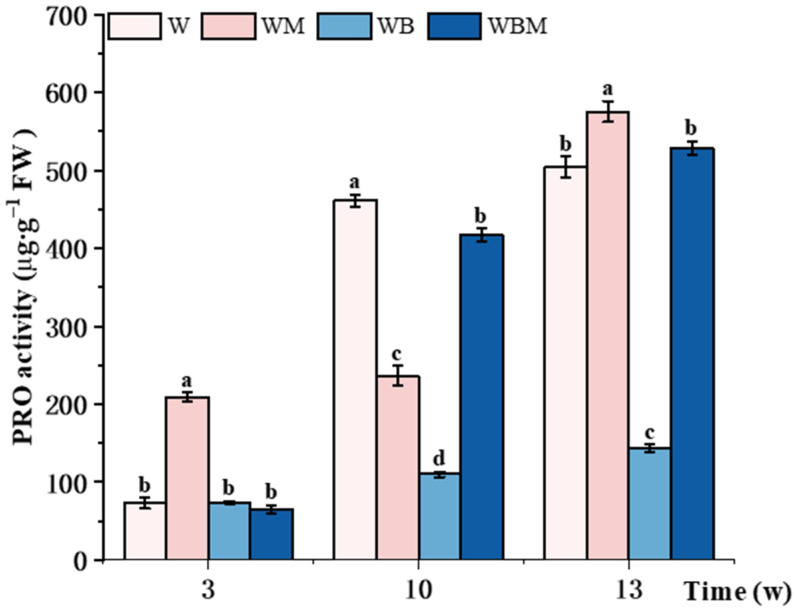
Changes in proline (PRO) content in sugar beet seedlings under different treatments. Note: Different lowercase letters in the figure indicate the significant differences (*p* < 0.05) between the different treatments. Vertical bars represent the standard error of means (±S.E.). Within each treatment, proline content differed significantly among the three time points (*p* < 0.05).

The changes in proline content are shown in Figure 8. At week 3, WM was highest (209.11 μg·g^−1^ FW), significantly exceeding W (72.92), WB (73.68), and WBM (64.89), which did not differ significantly (F_1,8_ = 520.67, *p* < 0.001; WM > W ≈ WB ≈ WBM). At week 10, the W group increased to 461.08 μg·g^−1^ FW, significantly exceeding WBM (417.19), WM (236.58), and WB (110.05), with the order W > WBM > WM > WB (F_1,8_ = 2789.46, *p* < 0.001). At week 13, WM was highest (575.42 μg·g^−1^ FW), significantly exceeding W (503.94), WBM (528.39), and WB (143.13); WBM and W did not differ but both exceeded WB (F_1,8_ = 636.68, *p* < 0.001; WM > WBM ≈ W > WB). In summary, blue light alone (WB) suppressed proline accumulation at all stages. MeJA promoted proline accumulation in a light quality-dependent manner: rapidly under white light (WM, week 3) but with a pronounced delay under blue light (WBM). By week 13, proline levels in the W, WM, and WBM groups all exceeded 500 μg·g^−1^ FW.

### 2.5. Correlation Analysis Among Physiological Parameters

**Figure 9:**
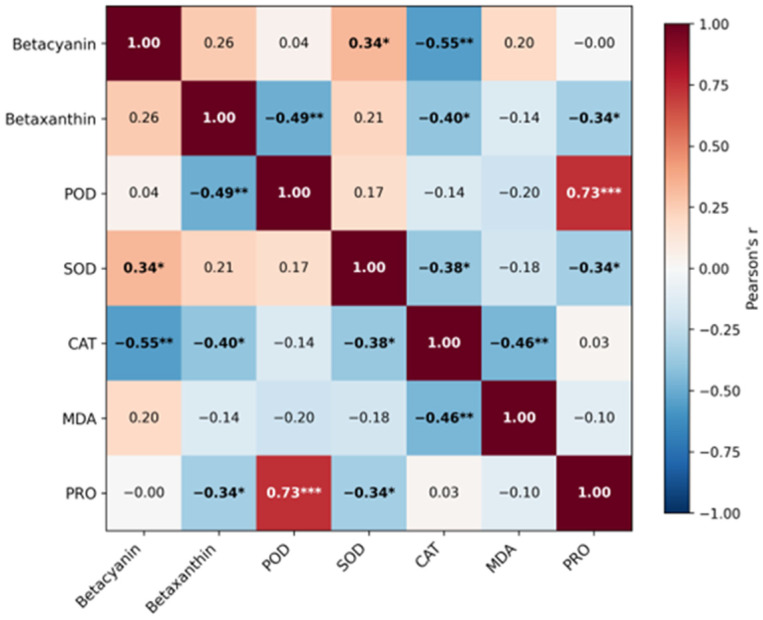
Pearson correlation heatmap among physiological parameters in sugar beet seedlings. The color scale represents Pearson’s correlation coefficient (r), with red indicating positive correlations and blue indicating negative correlations. Asterisks denote statistical significance (* *p* < 0.05, ** *p* < 0.01, *** *p* < 0.001). Data from all four treatments (W, WM, WB, WBM) and three time points (weeks 3, 10, and 13) were pooled (n = 36). Abbreviations: Betacyanin, betacyanin content; Betaxanthin, betaxanthin content; POD, peroxidase activity; SOD, superoxide dismutase activity; CAT, catalase activity; MDA, malondialdehyde content; PRO, proline content.

Pearson correlation analysis was performed among all seven measured parameters across all treatments and time points (n = 36) (Figure 9). The strongest positive correlation was between POD activity and proline content (r = 0.734, *p* < 0.001). Significant negative correlations included CAT activity with betacyanin (r = −0.547, *p* < 0.001), POD activity with betaxanthin (r = −0.492, *p* = 0.002), and CAT activity with MDA (r = −0.458, *p* = 0.005). Betaxanthin was also negatively correlated with proline (r = −0.342, *p* = 0.041).

Notably, the proline–MDA relationship exhibited a marked temporal shift. At week 3, the correlation was weak and non-significant (r = −0.244, *p* = 0.446). At week 10, a significant positive correlation emerged (r = 0.793, *p* = 0.002), indicating that higher proline levels accompanied higher MDA levels during the mid-growth stage. At week 13, the correlation inverted to significantly negative (r = −0.737, *p* = 0.006), suggesting that at the late stage, elevated proline was associated with reduced membrane lipid peroxidation. This temporal reversal was further modulated by treatment: proline and MDA were negatively correlated under W (r = −0.923, *p* < 0.001), positively correlated under WB (r = 0.958, *p* < 0.001), and not significantly correlated under WBM (r = −0.516, *p* = 0.156).

## 3. Discussion

This study revealed a clear temporal division of labor between blue light and MeJA in regulating betalain accumulation and the antioxidant system in sugar beet seedlings. Blue light alone (WB) acted as an early priming signal, rapidly inducing betaxanthin accumulation and SOD and POD activities while suppressing MDA at week 3. MeJA alone (WM) functioned as a mid-term burst elicitor, triggering peak betacyanin accumulation at week 10, accompanied by broadly enhanced antioxidant enzyme activities. The combined treatment (WBM) provided superior maintenance of both betacyanin and betaxanthin contents at week 13, supported by a significant light × MeJA interaction. Accordingly, we propose a temporal model in which blue light primes early antioxidant responses, MeJA drives mid-term pigment biosynthesis, and their combination sustains late-stage pigment levels. Similar light–hormone synergies have been reported in apple [28], mung bean [27], suggesting that temporal synergistic strategies may have broad significance in plant secondary metabolism.

Sugar beet differs from the model species used in comparable light–hormone studies in several fundamental respects. First, sugar beet accumulates betalains rather than anthocyanins. This mutual exclusion is underpinned by relaxed feedback regulation of the upstream tyrosine pathway [32], loss of transactivation function in anthocyanin-regulating MYB transcription factors [33], and repeated loss of the anthocyanidin synthesis enzyme TT19 with concomitant degeneration of the MBW (MYB–bHLH–WD40) regulatory complex [34]. Consequently, light- and jasmonate-induced pigment regulation in sugar beet—likely operating through MYC2 and HY5 to modulate CYP76AD and DODA family genes—differs fundamentally from the MBW-centered regulation in anthocyanin-accumulating species such as apple, grape, and Arabidopsis [30,31]. Direct mechanistic extrapolations between these systems should therefore be made with caution. Second, sugar beet is a biennial root crop whose taproot serves as the dominant carbon and nitrogen sink, in contrast to foliar or fruit crops. This sink–source configuration may influence secondary metabolite partitioning, potentially explaining the pronounced late-stage pigment maintenance under WBM. Third, cultivars such as HZTC11H constitutively accumulate high betalain levels in vegetative tissues, which may pre-condition the cellular redox environment and alter the threshold for elicitor-triggered antioxidant responses.

The two betalain types showed divergent treatment sensitivities. Betacyanin synthesis requires CYP76AD1, which possesses both tyrosine hydroxylase and cyclo-DOPA-forming activities, whereas betaxanthin requires only the monooxygenase activity of CYP76AD5/6 [11]. Blue light may preferentially upregulate CYP76AD5/6 via HY5, while MeJA may activate CYP76AD1 through MYC2, enabling branch-specific temporal regulation [35]. Supporting this, blue light upregulates CYP76AD1 and DODA expression in amaranth while increasing betalain content approximately 2.3-fold [36], and the AtrDODA1-1 promoter contains MeJA-responsive elements that drive DODA expression upon MeJA treatment [37].

The three antioxidant enzymes exhibited clear temporal coordination. SOD activity predominated at the early stage, whereas POD and CAT increased progressively, peaking at week 13, consistent with the sequential scavenging of superoxide anions followed by H_2_O_2_. Treatment effects were enzyme-specific: MeJA strongly activated CAT early and POD and SOD in the mid-to-late stages; blue light activated SOD and POD early and maintained CAT at intermediate levels. When CAT was suppressed (WM, week 13), POD increased compensatorily, and vice versa, indicating functional redundancy among these enzymes. This plasticity enables flexible antioxidant resource allocation and is consistent with temporal patterns observed in wheat and grape under MeJA treatment [38,39].

The proline–MDA relationship exhibited a striking temporal reversal. At week 10, proline and MDA were positively correlated (r = 0.793, *p* = 0.002), suggesting that proline accumulation parallels oxidative stress during active growth. At week 13, the correlation inverted to negative (r = −0.737, *p* = 0.006), indicating a functional transition of proline from a general stress indicator to a protective osmolyte. This relationship was strongly modulated by treatment: a negative correlation under W (r = −0.923, *p* < 0.001) contrasted with a positive correlation under WB (r = 0.958, *p* < 0.001), while MeJA-containing treatments showed non-significant correlations (WM: r = 0.446; WBM: r = −0.516), suggesting that MeJA partially decouples proline accumulation from membrane oxidative status. The WM treatment maintained high proline and low MDA throughout all stages, confirming that exogenous MeJA rapidly and persistently induces proline accumulation, as also reported in grape and wheat [38,39]. However, under a blue light background (WBM), the MeJA effect was delayed: early-stage proline was even lower than WB, and MDA was nearly double, suggesting that blue light may transiently interfere with MeJA signal transduction [31]. By the late stage, proline in WBM had increased and MDA declined to levels comparable to WM, indicating adaptive restoration of MeJA responsiveness. The molecular basis of this lag effect may involve the temporal dynamics of MYC2–HY5 signal integration [30,31], although direct experimental validation in sugar beet is required.

The pre-harvest elicitation strategy described here represents one of several available betalain production platforms. Microbial fermentation in engineered *Yarrowia lipolytica* achieved a betanin titer of 1271 mg·L^−1^ with reduced environmental impact compared to conventional extraction [40]. Hairy root cultures and cell suspension cultures offer continuous, controlled production but require significant bioreactor infrastructure [41]. Compared with these platforms, the whole-plant pre-harvest approach requires minimal infrastructure and is compatible with existing controlled-environment agriculture, though constrained by longer production cycles and environmental sensitivity [42]. These platforms may be considered complementary, each suited to distinct production contexts.

Although the pre-harvest whole-plant approach described here requires minimal infrastructure, its productivity is lower than that of microbial fermentation platforms [40] and hairy root bioreactor systems [41]; these platforms may be considered complementary [42]. This study has several limitations, including the absence of molecular validation, the use of a single cultivar, and the qualitative nature of growth assessments. These constraints should be considered when interpreting the findings.

## 4. Materials and Methods

### 4.1. Plant Material and Growth Conditions

The sugar beet (*Beta vulgaris* L.) cultivar used was “HZTC11H”, a red beet genotype maintained in our laboratory. This cultivar was selected from several laboratory-maintained red beet genotypes based on its superior root health during prolonged cultivation. Seeds were surface-sterilized in 75% ethanol for 30 s, followed by 2% sodium hypochlorite for 10 min, rinsed three times with deionized water, and germinated in darkness at 25 °C. After the second pair of true leaves had fully expanded, uniformly grown seedlings were transplanted into plastic buckets (26 cm diameter, 27 cm height). Growth conditions were: 12 h light/12 h dark photoperiod, day/night temperatures of 23 °C and 18 °C, respectively. White light was provided by white LED fixtures (KING WUA BRIGHT Technology Co., Ltd., Shenzhen, China; 19-core, peak wavelength 444 nm, broad-spectrum emission with a color rendering index of 88.7), and blue light by blue LED fixtures (KING WUA BRIGHT Technology Co., Ltd., Shenzhen, China; 90-core, peak wavelength 453 nm, narrow-band emission with a full width at half maximum [FWHM] of 26.0 nm). The emission spectra of both light sources (Supplementary Figure S1) were measured at canopy height using a spectroradiometer (PLA-20, Everfine Corporation, Hangzhou, China). The photosynthetic photon flux density (PPFD) was maintained at 130 μmol·m^−2^·s^−1^ for all treatments, as measured with a quantum sensor (HPL-220P, Hopocolor, Shenzhen, China).

### 4.2. Light Quality and MeJA Treatments

After transplanting, seedlings were randomly divided into four groups of 30 buckets each. The treatments were as follows:

W (white light control): white LED light source;

WM (white light + MeJA): white light conditions + foliar spray of 150 μmol·L^−1^ MeJA;

WB (white + blue light): combined white and blue light source, with a photon flux ratio of 4:1 (white light accounting for 80% of total PPFD, blue light 20%);

WBM (white + blue light + MeJA): combined white + blue light source + foliar spray of 150 μmol·L^−1^ MeJA.

The MeJA concentration (150 μmol·L^−1^) was selected based on previous studies demonstrating that foliar application of 100–200 μmol·L^−1^ MeJA effectively induces betalain accumulation in *Alternanthera* spp. and *Celosia argentea* cell suspension cultures without visible phytotoxicity [24,26]. The white-to-blue photon flux ratio (4:1; blue light accounting for 20% of total PPFD) was chosen to provide a moderate blue light stimulus while avoiding the growth-inhibitory effects associated with higher proportions of blue light, with the total PPFD maintained at 130 μmol·m^−2^·s^−1^, consistent with levels commonly used for controlled-environment cultivation of beet seedlings [18].

Methyl jasmonate (MeJA, purity ≥ 98%, CAS No. 39924-52-2) was purchased from Aladdin Biochemical Technology Co., Ltd. (Shanghai, China; Catalog No. M111206). MeJA was dissolved in absolute ethanol to prepare a 100 mM stock solution, then diluted to 150 μmol·L^−1^ with distilled water (final ethanol concentration: 0.1% *v*/*v*). Tween-20 (0.02% *v*/*v*) was added as a surfactant to ensure uniform leaf wetting. The solution was freshly prepared before each application and applied as a foliar spray until runoff using a handheld sprayer. Control groups (W and WB) were sprayed with an equivalent volume of 0.1% ethanol aqueous solution.

Sampling was performed at weeks 3, 10, and 13 after treatment. Six buckets were randomly selected per treatment, and 3–4 fully expanded leaves were taken from each bucket. Samples from the same treatment were pooled, frozen immediately in liquid nitrogen, and stored at −80 °C until analysis.

### 4.3. Determination of Betacyanin and Betaxanthin Contents

Approximately 0.25 g of frozen sample was ground to a fine powder in liquid nitrogen. Extraction was performed by adding 1 mL of 60% methanol (containing 0.1% ascorbic acid) at a solid-to-liquid ratio of 1:4 (*w*/*v*), vortexed for 2 min, and extracted overnight at 4 °C. The extract was centrifuged at 13,000× *g* for 15 min at 4 °C, and the supernatant was collected. The extraction step was repeated once, and the two supernatants were combined, filtered through a 0.45 μm filter into a new centrifuge tube. Absorbance was measured at 538 nm for betacyanin and 470 nm for betaxanthin. Contents were calculated using the following formula:

(1) $$B\;(mg\cdot g^{-1})\;=\;(A\;\times \;DF\;\times \;W\;\times \;V\;\times \;100)/(\varepsilon \;\times \;P)$$

*B* (mg·g^−1^ FW) = (*A* × *DF* × *W* × *V* × 100)/(*ε* × *P*)
*A*: absorbance value
*DF*: dilution factor
*W*: molecular weight (550 g·mol^−1^ for betacyanin; 308 g·mol^−1^ for betaxanthin)
*V*: solution volume (mL)
*ε*: extinction coefficient (60,000 L/mol·cm for betacyanin; 48,000 L/mol·cm for betaxanthin)
*P*: fresh weight (g)

### 4.4. Determination of Antioxidant Enzyme Activities

Frozen sample (approximately 0.1 g) was mixed with 1 mL of extraction buffer (provided by the kit), homogenized on ice, and centrifuged at 8000× *g* for 10 min at 4 °C. The supernatant was collected as the crude enzyme extract and kept on ice. All assays were performed using commercial kits from Suzhou Comin Biotechnology Co., Ltd. (Suzhou, China): POD (catalog No. POD-1-Y), SOD (catalog No. SOD-1-Y), CAT (catalog No. CAT-1-Y), MDA (catalog No. MDA-1-Y), and PRO (catalog No. PRO-1-Y). All assays were performed in 96-well microplates with three technical replicates per sample.

#### 4.4.1. POD Activity Assay

A microplate method was used, based on the principle that POD catalyzes the oxidation of a specific substrate by H_2_O_2_, resulting in a characteristic light absorption at 470 nm. The microplate reader was preheated for 30 min and zeroed. The working solution was prepared by mixing reagents 1, 2, and 3 (2.6 mL: 1.5 μL: 1 μL) and preheated at 25 °C for 10 min. In a 96-well plate, 10 μL of crude enzyme extract and 190 μL of working solution were added, mixed, and the absorbance was immediately measured at 470 nm at 1 min (A1) and 2 min (A2). Δ*A* = A2 − A1 was calculated. *POD* activity was calculated as:

(2) $$POD\;(U\cdot g^{-1}\;FW)\;=\;2000\;\times \;\Delta A/W$$

*W*: sample fresh weight (g)

#### 4.4.2. SOD Activity Assay

A microplate method was used, based on the principle that SOD scavenges superoxide anions and inhibits formazan formation, with activity calculated by measuring the inhibition rate of absorbance at 560 nm. After preheating the microplate reader for 30 min, reagent 2 was diluted 1:1 with distilled water, and reagent 4 was dissolved in 5 mL distilled water and then diluted 1:3 with distilled water. In a 96-well plate, 45 μL of reagent 1, 2 μL of reagent 2, 18 μL of distilled water (for the assay tube, 18 μL of crude enzyme extract; for the control tube, 18 μL of distilled water), and 35 μL of reagent 3 were added, mixed thoroughly, and allowed to stand at room temperature for 30 min. Absorbance at 560 nm was measured for the assay tube (*A_assay*) and control tube (*A_control*). The inhibition percentage was calculated as:

(3) Inhibition (%) = (A_control − A_assay)/A_control × 100

SOD activity was calculated as:

SOD (U·g^−1^ FW) = 11.11 × *Inhibition* (%)/(1 − *Inhibition* (%)) ÷ *W*
*W*: sample fresh weight (g)

#### 4.4.3. CAT Activity Assay

A microplate method was used, based on the principle that CAT decomposes H_2_O_2_, causing a decrease in absorbance at 240 nm over time. A UV spectrophotometer or microplate reader equipped with UV plates was used. After preheating for 30 min and zeroing, the CAT detection working solution was preheated in a 25 °C water bath for at least 10 min. In a 96-well UV plate, 10 μL of crude enzyme extract and 190 μL of working solution were added, mixed, and the absorbance at 240 nm was immediately measured at the initial time (A1) and after 1 min (A2). Δ*A* = A1 − A2 was calculated. *CAT* activity was calculated as:

(4) $$CAT\;(U\cdot g^{-1}\;FW)\;=\;918\;\times \;\Delta A/W$$

*W*: sample fresh weight (g)

### 4.5. Determination of Malondialdehyde (MDA) and Proline (PRO) Contents

#### 4.5.1. MDA Content Assay

A microplate method was used with a malondialdehyde (MDA) content assay kit (Suzhou Comin Biotechnology Co., Ltd., catalog No. MDA-1-Y). Approximately 0.1 g of frozen sample was mixed with 1 mL of extraction buffer, homogenized on ice, and centrifuged at 8000× *g* for 10 min at 4 °C. The supernatant was collected as the test solution. In a 1.5 mL centrifuge tube, 270 μL of reagent 1 (if not completely dissolved before use, heat and shake at 70–90 °C to aid dissolution) and 90 μL of test solution were added, mixed, capped tightly, and incubated in a 95 °C water bath for 30 min, then cooled in an ice bath and centrifuged at 10,000× *g* for 10 min at 25 °C. An aliquot of 200 μL of the supernatant was transferred to a 96-well plate, and the absorbance at 532 nm and 600 nm was measured. Δ*A* = A_532_ − A_600_ was calculated. *MDA* content was calculated as:

(5) $$MDA\;(nmol\cdot g^{-1}\;FW)\;=\;51.6\;\times \;\Delta A/W$$

*W*: sample fresh weight (g)

#### 4.5.2. PRO Content Assay

A microplate method was used with a proline (PRO) content assay kit (Suzhou Comin Biotechnology Co., Ltd., catalog No. PRO-1-Y). Approximately 0.1 g of frozen sample was mixed with 1 mL of extraction buffer, homogenized on ice, and then extracted in a 95 °C water bath with oscillation for 10 min, followed by centrifugation at 10,000× *g* for 10 min at 25 °C. The supernatant was collected as the test solution. In an EP tube, 0.25 mL of test solution, 0.25 mL of glacial acetic acid (reagent 1), and 0.25 mL of reagent 2 were added sequentially, capped tightly, and incubated in a 95 °C water bath for 30 min (with oscillation every 10 min). After cooling, 0.5 mL of toluene (reagent 3) was added, shaken for 30 s, and allowed to stand to transfer the red pigment to the upper organic phase. An aliquot of 0.2 mL of the upper layer was transferred to a 96-well plate, and the absorbance at 520 nm was measured. Proline content was calculated as:

(6) $$PRO\;(\mu g\cdot g^{-1}\;FW)\;=\;38.4\;\times \;(A\;+\;0.0021)/W$$

*W*: sample fresh weight (g)

### 4.6. Statistical Analysis

All experiments were performed with three biological replicates, and data are expressed as means ± standard deviations (SDs). Normality within each treatment group was assessed using the Shapiro–Wilk test, and homogeneity of variance was verified using Levene’s test. Normality was confirmed (*p* > 0.05) in 18 of 21 indicator × time point combinations. In three instances where minor normality deviations were detected in a single treatment group (CAT at week 13, proline at week 3, and betaxanthin at week 13), Levene’s test confirmed variance homogeneity (*p* > 0.05), and the non-parametric Scheirer–Ray–Hare test was applied as validation, confirming the same significance levels for the interaction term. For all datasets, the effects of light quality (W vs. WB), MeJA treatment (0 vs. 150 μM), and their interaction were analyzed by two-way ANOVA, followed by Tukey’s HSD post hoc test for multiple comparisons. Pearson correlation coefficients were calculated using SPSS 21.0 (SPSS Inc., Chicago, IL, USA). A significance level of *p* < 0.05 was adopted for all tests. Figures were generated using GraphPad Prism 8.0 (GraphPad Software, San Diego, CA, USA).

## 5. Conclusions

This study revealed a temporal synergistic pattern by which blue light and methyl jasmonate (MeJA) regulate betalain accumulation and antioxidant defense in sugar beet seedlings. WB acted as an early priming signal, rapidly inducing betaxanthin accumulation and SOD and POD activities while suppressing lipid peroxidation at week 3. MeJA alone (WM) functioned as a mid-term burst elicitor, triggering peak betacyanin accumulation (45.74 mg·g^−1^ FW) at week 10 and broadly enhancing antioxidant enzyme activities. The combined treatment (WBM) provided superior maintenance of both betacyanin and betaxanthin contents at the late stage (week 13), supported by a statistically significant light × MeJA interaction. However, this combined effect was specific to pigment accumulation; for individual antioxidant enzymes—particularly CAT—and proline accumulation, single-factor treatments were equally or more effective at certain time points. The three antioxidant enzymes exhibited temporal division of labor, with SOD predominating early and POD and CAT increasing progressively, alongside functional compensation among them. A temporal reversal in the proline–MDA correlation—from positive at week 10 (r = 0.793, *p* = 0.002) to negative at week 13 (r = −0.737, *p* = 0.006)—further indicated that proline transitions from a general stress indicator to a protective osmolyte during late-stage development, a shift that was modulated by both light quality and MeJA treatment. These findings demonstrate that blue light and MeJA enhance pigment yield and antioxidant capacity through coordinated temporal regulation, offering a potential preharvest strategy for natural pigment production in controlled environments.

## Data Availability

The data presented in this study are available from the corresponding author upon reasonable request.

## Supplementary Materials

The following supporting information can be downloaded at: https://www.mdpi.com/article/10.3390/plants15131994/s1, Figure S1: Emission spectra of the white LED (A) and blue LED (B) used in this study.

## Abbreviations

The following abbreviations are used in this manuscript:

MeJA	Methyl Jasmonate
POD	Peroxidase
SOD	Superoxide Dismutase
CAT	Catalase
MDA	Malondialdehyde
PRO	Proline
