# Supplementary material for: Blue Light and Methyl Jasmonate Synergistically Enhance Betalain Accumulation, Antioxidant Enzyme Activity, and Osmotic Adjustment in Sugar Beet (Beta vulgaris L.) Seedlings: A Time-Course Analysis
Source: Plants (Basel). 2026 Jun 27;15(13):1994. doi: 10.3390/plants15131994 (PMC13364101; doi:10.3390/plants15131994)
Supplement: Supplementary file 1 [file plants-15-01994-s001.zip › plants-4331081-supplementary.pdf]

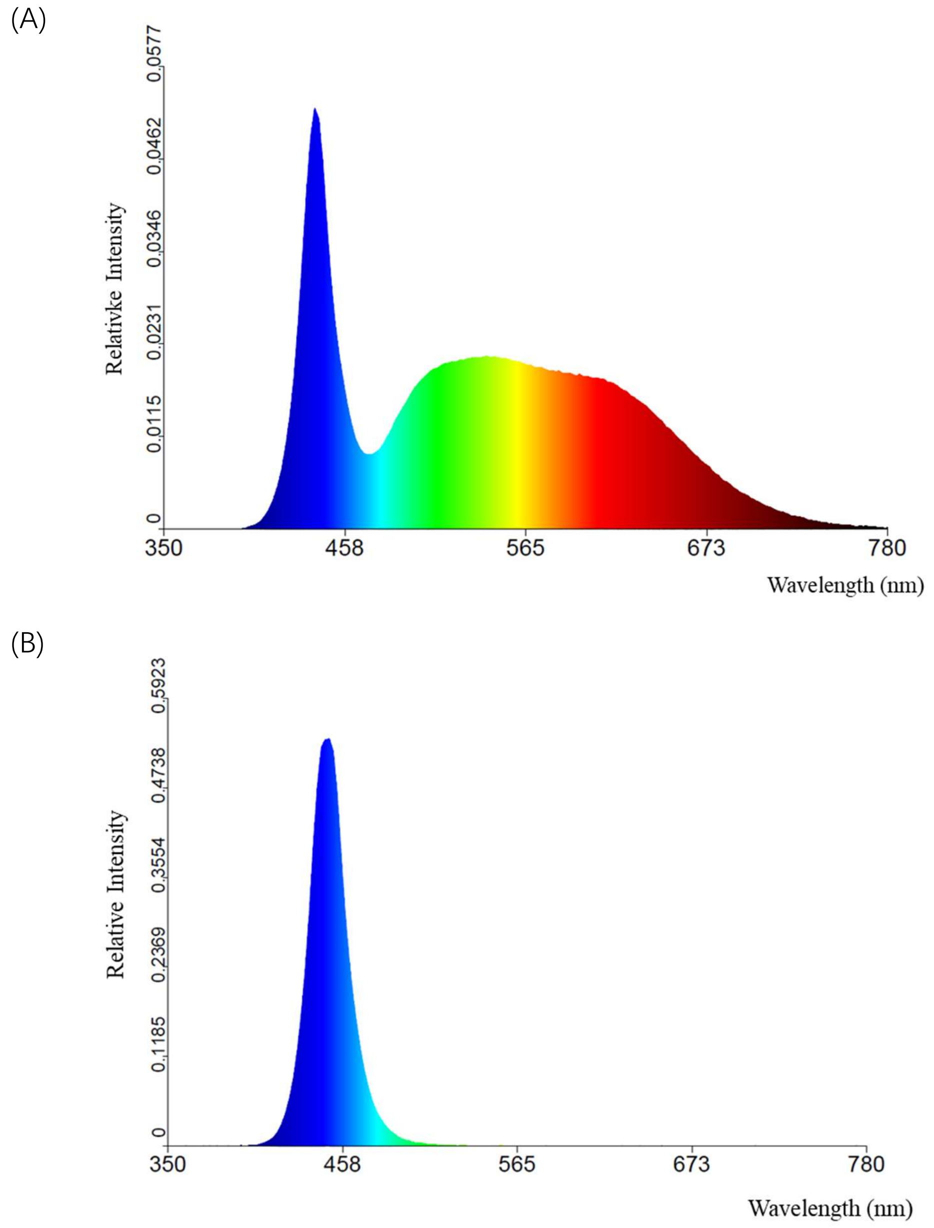

Figure. S1. Emission spectra of the white LED (A) and blue LED (B) used in this study. Spectral distributions were measured at canopy height using a spectroradiometer (PLA-20, Everfine Corporation, Hangzhou, China). The white LED (A) exhibited a broad-spectrum emission covering the entire visible range, with a peak wavelength at 444 nm and a color rendering index (CRI Ra) of 88.7, characteristic of a phosphor-converted white LED. The blue LED (B) showed a narrow-band emission centered at 453 nm, with a full width at half maximum (FWHM) of 26.0 nm, producing near-monochromatic blue light. Spectra are normalized to their respective maximum values for direct comparison of spectral shape.
